# Silencing Prion Protein in MDA-MB-435 Breast Cancer Cells Leads to Pleiotropic Cellular Responses to Cytotoxic Stimuli

**DOI:** 10.1371/journal.pone.0048146

**Published:** 2012-11-02

**Authors:** Guohua Yu, Liming Jiang, Yuanyuan Xu, Hongwei Guo, Huiyan Liu, Yi Zhang, Huaiyi Yang, Chonggang Yuan, Jiyan Ma

**Affiliations:** 1 School of Life Sciences, Key Laboratory of Brain Functional Genomics, Ministry of Education, Shanghai Key Laboratory of Brain Functional Genomics, East China Normal University, Shanghai, China; 2 Department of Molecular and Cellular Biochemistry, Ohio State University, Columbus, Ohio, United States of America; 3 CAS Key Laboratory of Pathogenic Microbiology and Immunology, Institute of Microbiology, Chinese Academy of Sciences, Beijing, China; The Scripps Research Institute Scripps Florida, United States of America

## Abstract

Prion protein (PrP) is well studied for its pathogenic role in prion disease, but its potential contribution to other pathological processes is less understood. PrP is expressed in a variety of cancers and at least in pancreatic and breast cancers, its expression appears to be associated with poor prognosis. To understand the role of PrP in breast cancer cells, we knocked down PrP expression in MDA-MB-435 breast cancer cells with small interfering RNA and subjected these cells to a series of analyses. We found that PrP knockdown in these cells does not affect cell proliferation or colony formation, but significantly influences the cellular response to cytotoxic stimuli. Compared to control cells, PrP knockdown cells exhibited an increased susceptibility to serum deprivation induced apoptosis, no change to staurosporine- or paclitaxel-induced cell deaths, and a reduced susceptibility to chemotherapy drug doxorubicin-induced cell death. To understand the mechanism of unexpected role of PrP in exacerbating doxorubicin-induced cytotoxicity, we analyzed cell death related Bcl-2 family proteins. We found that PrP knockdown alters the expression of several Bcl-2 family proteins, correlating with increased resistance to doxorubicin-induced cytotoxicity. Moreover, the enhanced doxorubicin resistance is independent of DNA damage related p53 pathway, but at least partially through the ERK1/2 pathway. Together, our study revealed that silencing PrP in MDA-MB-435 breast cancer cells results in very different responses to various cytotoxic stimuli and ERK1/2 signaling pathway is involved in PrP silencing caused resistance to doxorubicin.

## Introduction

Prion protein (PrP) is a glycosylphosphatidylinositol (GPI)-anchored cell surface glycoprotein, which is widely expressed in various tissues with most abundant expression in the central nervous system. Because of its critical role in the pathogenesis of transmissible spongiform encephalopathies (also known as “prion disease”), PrP has been investigated extensively. It has been shown that an aberrantly folded PrP isoform is the infectious agent in prion disease and the expression of normal PrP is essential for neurodegeneration [1], [2], [3], [4]. Besides its role in prion disease, normal PrP expression has been found to contribute to many important biological processes, such as cell adhesion, neurite outgrowth, synaptic transmission, oxidative stress, cell survival, etc [5], [6], [7]. But thus far, the precise physiological function of PrP remains unclear.

Several groups have reported that PrP expression is up-regulated in a variety of human cancers, including gastric carcinoma [8], [9], osteosarcoma [10], breast cancer [11], melanoma [12], and pancreatic cancer [13]. More importantly, at least two studies revealed that PrP expression is associated with poor prognosis in pancreatic and breast cancers [11], [13], suggesting a contributory role of PrP in cancer biology. Indeed, PrP has been found to enhance cancer cell proliferation, metastasis, and resistance to cell death [14], which are generally consistent with the pro-survival, anti-stress, and promoting cell adhesion properties of PrP found in neuronal cells [5], [6]. Various cellular mechanisms have been proposed to explain the role of PrP in cancer cells, including the activation of PI3K/Akt signaling pathway to up-regulate cyclin D in gastric cancer cells [9], chemotherapy drug induced PrP interaction with P-glycoprotein (P-gp, ATP-dependent drug-efflux pumps ABCB1) in a drug-resistant MCF7 breast cancer subline [15], and the presence of an aberrantly processed pro-PrP form that disrupts normal cell physiology by binding to filamin A in melanoma and pancreatic cancer cells [12], [13].

The contribution of PrP to breast cancer biology has been shown by several studies [11], [16], [17], [18]. It was reported that over-expression of PrP in MCF7 breast cancer cells inhibit tumor necrosis factor alpha (TNF)- or Bax-induced cell death [16], [18]. Silencing PrP in drug-resistant MCF7 sublines sensitizes these cells to tumor necrosis factor-related apoptosis inducing ligand (TRAIL)- or chemotherapeutic drug paclitaxel-induced cell death [15], [17]. More importantly, PrP expressing estrogen receptor (ER)-negative breast cancers appear to respond poorly to adjuvant chemotherapy (chemotherapy after surgery) [11]. Thus far, breast cancer cell-based studies have used the approaches of overexpressing PrP in breast cancer cells or silencing PrP in selected drug-resistance cell sublines. It remains unclear whether PrP knockdown is able to enhance chemotherapy drug induced toxicity in breast cancer cells that have not been pre-selected by drug-resistance.

In this study, we knocked down PrP expression in estrogen receptor-negative breast cancer MDA-MB-435 cell line with a retrovirus-based RNA interference system. Our results showed that PrP plays very different roles in response to various cytotoxic stimuli.

## Materials and Methods

### Construction of retrovirus-based RNAi plasmid

The shRNA oligonucleotide corresponding to human *PRNP* sequences 627–645 (5′-GGTTGAGCAGATGTGTATC-3′) was synthesized and cloned into the down-stream of U6 promoter in the pSIREN-RetroQ vector (Clontech Lab., Inc.), named as pSIREN-RetroQ-siPrP. A control vector named pSIREN-RetroQ-siLuc was constructed, which carries shRNA targeting to luciferase and does not affect gene expression in human cells.

### Cell culture

The MDA-MB-435 cell line was obtained from American Type Culture Collection. Both MDA-MB-435 breast cancer cells and phoenix cells (recombinant retrovirus packaging cells) were maintained in Dulbecco's modified Eagle's medium (DMEM) supplemented with 10% fetal bovine serum, streptomycin (100 µg/mL) and penicillin 100 units/mL. Cells were cultured at 37°C in a humidified incubator with 5% CO_2_.

### Production of recombinant retrovirus

Around 5×10^5^ phoenix packaging cells in a 60 mm cell culture dish were transfected with 2.5 µg of recombinant construct using GenJet™ In Vitro DNA Transfection Reagent (SignaGen Laboratories). Forty-eight hours after transfection, the supernatant containing recombinant retrovirus was collected, filtered through a 0.45 µm syringe filter and used to infect target cells.

### Recombinant retrovirus infection of MDA-MB-435 cells

One day before transduction, 1×10^6^ MDA-MB-435 cells per well were plated in 60 mm cell culture dish. On the next day, the recombinant retrovirus containing supernatant with 8 µg/mL polybrene was added to the cells. After 24 hours, the cells were re-plated at lower density and selected by using medium containing 0.25 µg/mL puromycin. The puromycin selection was continued for 2 weeks and all the resistant cell colonies from one recombinant viral infection were pooled together and continue to culture as a stable cell line. This bulk-selected stable cell lines were used for all the experiments except for those indicated. The stable cell line infected with pSIREN-RetroQ-siPrP virus was named as siPrP cell line, while the stable cell line infected with pSIREN-RetroQ-siLuc virus was named as siLuc cell line.

### Establishment of siPrP-HA-p53 and siLuc-HA-p53 stable cell lines

The siPrP-HA-p53 or siLuc-HA-p53 stable cell lines were established by transfecting siPrP cells or siLuc cells with pcDNA3.1-HA-p53 plasmid. Transfected cells were selected with 2 mg/mL G418 for two weeks. All G418-resistant cell colonies were pooled together and used for analyses.

### Immunoblot analysis

Cells were scraped and homogenized in lysis buffer containing 50 mM Tris-HCl, pH7.5, 150 mM NaCl, 5 mM EDTA containing 0.5% Triton X-100 and 0.5% sodium deoxycholate. Equal amounts of protein (40 µg) were separated on sodium dodecyl sulfate polyacrylamide gel electrophoresis (SDS-PAGE) and transferred to polyvinylidene fluoride (PVDF) membranes. After incubation in blocking buffer (50 mM Tris-HCl, pH 7.5, 150 mM NaCl, 5% non-fat dry milk) for one hour at room temperature, membranes were incubated in the same buffer containing a primary antibody for two hours at room temperature. Primary antibodies were: PrP (SAF32, Cayman); p53, HA-tag, active caspase-3, PARP, Bcl-2, Bax, Total-ERK1/2 (Cell Signaling Technology); beta-actin (Sigma); A1, Bcl-XL, PUMA, NIP3 (Epitomics); Bcl-W, BMF, Mcl-1, BID, BAD (Proteintech Group); Bak (Bioworld Technology); phospho-ERK1/2 (Milipore). After incubation with primary antibody, membranes were washed and incubated for one hour in the blocking buffer containing horseradish peroxidase-conjugated goat anti-mouse IgG or horseradish peroxidase-conjugated goat anti-rabbit IgG antibody (Bio-Rad). Membranes were developed using Immobilon Western Chemiluminescent HRP Substrates (Millipore) and visualized by using a ChemiDoc XRS system (Bio-Rad).

### Immunofluorescent assay and endogenous fluorescence of doxorubicin

Cells were cultured on coverslip in 24-well plate overnight, rinsed three times with PBS and then fixed in 4% paraformaldehyde for 30 min at room temperature. PrP was detected with 1∶100 dilution of mouse monoclonal anti-PrP 3F4 antibody (Covance Inc.) and 1∶200 dilution of Alexa 488 conjugated goat anti-mouse IgG antibody (Invitrogen). Nuclei were stained with 4′,6-diamidino-2-phenylindole (DAPI). The immunofluorescence or endogenous fluorescence of doxorubicin was visualized with a microscope (Leica) and images were taken by a Leica DFC310FX digital camera.

### Cell viability

Cell viability was measured with MTT assay as previously described [19]. Briefly, 1×10^4^ cells per well were plated into a 96-well cell culture plate. All treatments were performed after cells were cultured for 24 hours. After indicated treatments, 3-(4,5-dimethylthiazol-2-yl)-2,5-diphenyltetrazolium-bromide (MTT, Sigma) was added to reach a final concentration 0.5 mg/mL and incubated for 4 hours at 37°C. After removing the media, 100 µL DMSO was added and plates were read at 570 nm on a micro ELISA plate reader. Cell viability was expressed as a percentage of untreated cells. Cell growth curve was determined by essentially the same method as cell viability determination, except that 2×10^3^ cells per well were plated and cell proliferation was expressed as a percentage of day 0 cells viability. The following chemicals were used to treat cultured cells: doxorubicin (Sigma), paclitaxel, staurosporine, and PD98059 (Santa Cruz biotechnology, Inc.).

### Soft agar assay for colony formation

A total of 5×10^3^ siLuc or siPrP cells were seeded onto six-well plates containing 0.5% base agar and 0.35% top agarose with complete culture medium containing 10% fetal bovine serum. Cells were allowed to grow for 25 days at 37°C and 5% CO_2_. The number of colonies was counted after staining with 0.05% crystal violet for 1 hour.

### Trypan blue exclusion assay

Cells were cultured in 35 mm dishes and subjected to doxorubicin treatment. After the treatment, culture medium containing suspended cells was transferred to a 1.5 mL centrifuge tube. The adhered cells were detached by trypsin digestion and added to the same tube. After centrifugation at 1,000 rpm for 5 minutes, cells were re-suspended in 0.5 mL cell culture medium. Ten microliters cell suspension was mixed with 10 µL of 0.4% trypan blue, and 10 µL mixture was used for automatic counting in a TC10 Automated Cell Counter (Bio-Rad).

### Statistical analysis

Statistical analysis was performed with SigmaPlot 10.0 software. Results of MTT and the live/dead cell counting assay represent the average ± standard deviation of five independent samples. Each experiment was repeated for at least three times and all results were consistent. All results were analyzed by Student's t test and a *P* value <0.05 was considered statistically significant.

## Results

### PrP knockdown in MDA-MB-435 cells does not affect cell proliferation or colony formation

**Figure 1:**
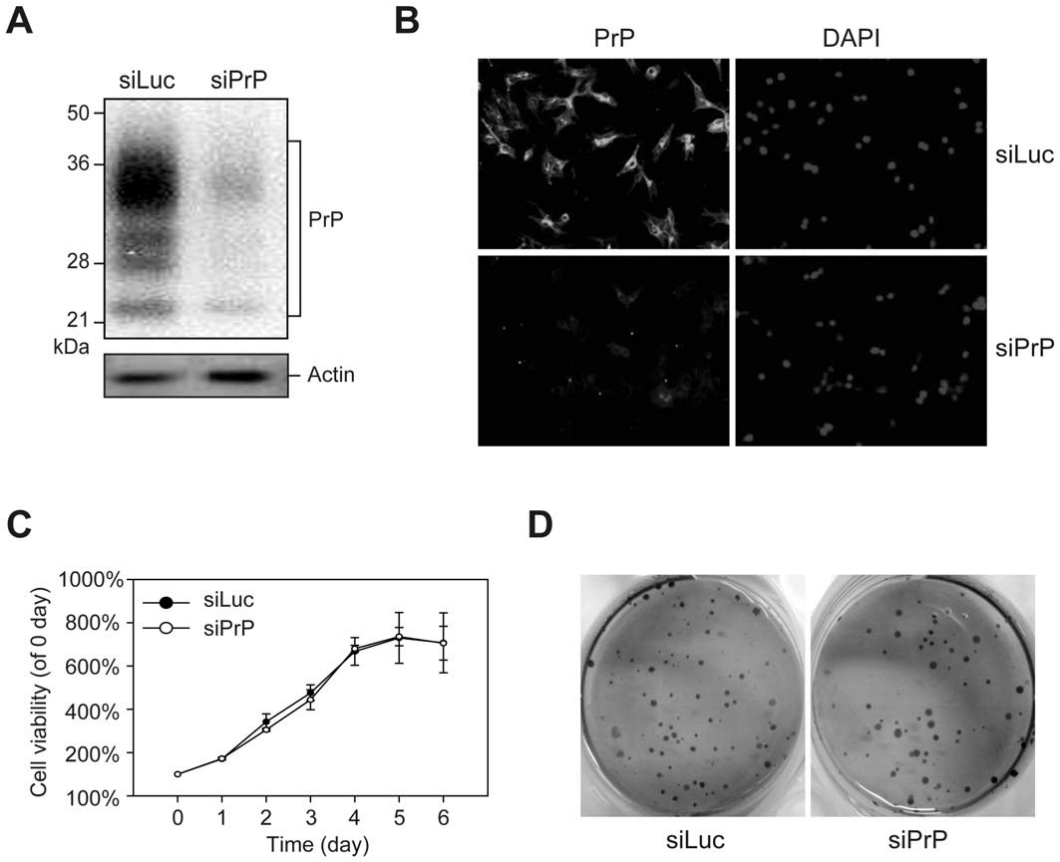
Effects of PrP knockdown on cell proliferation and colony formation. Levels of PrP expression in control siLuc cells (siLuc) and PrP knockdown cells (siPrP) was determined by immunoblot (**A**) and immunofluorescence analyses (**B**). Nuclei were stained with DAPI as indicated. (**C**) Proliferation curves of siPrP and siLuc cells were determined by the MTT assay. (**D**) Representative images of soft agar colony formation assay of siLuc and siPrP cells as indicated.

Human breast cancer MDA-MB-435 cells were infected with retrovirus expressing a short hairpin RNA (shRNA) corresponding to human *PRNP* sequences 627–645. To generate a control cell line, MDA-MB-435 cells were also infected with retrovirus expressing an shRNA against luciferase. Infected cells were bulk-selected with puromycin and the stable lines were named as siPrP and siLuc, respectively. The efficiency of PrP knockdown was verified by immunoblot and immunofluorescence analyses, demonstrating a significant reduction of PrP expression in siPrP cells (Fig. 1A and B).

The influence of PrP knockdown on cell proliferation and anchorage-independent cell growth were analyzed by MTT and soft agar colony formation assay. Figure 1C showed that the presence or absence of PrP expression does not affect MDA-MB-435 cell proliferation and the growth curves for siPrP and siLuc cells were almost identical. Similarly, soft agar assay revealed that PrP knockdown does not affect the colony formation capability of MDA-MB-435 cells (Fig. 1D).

### The effects of PrP knockdown on cellular response to various cytotoxic stimuli

**Figure 2:**
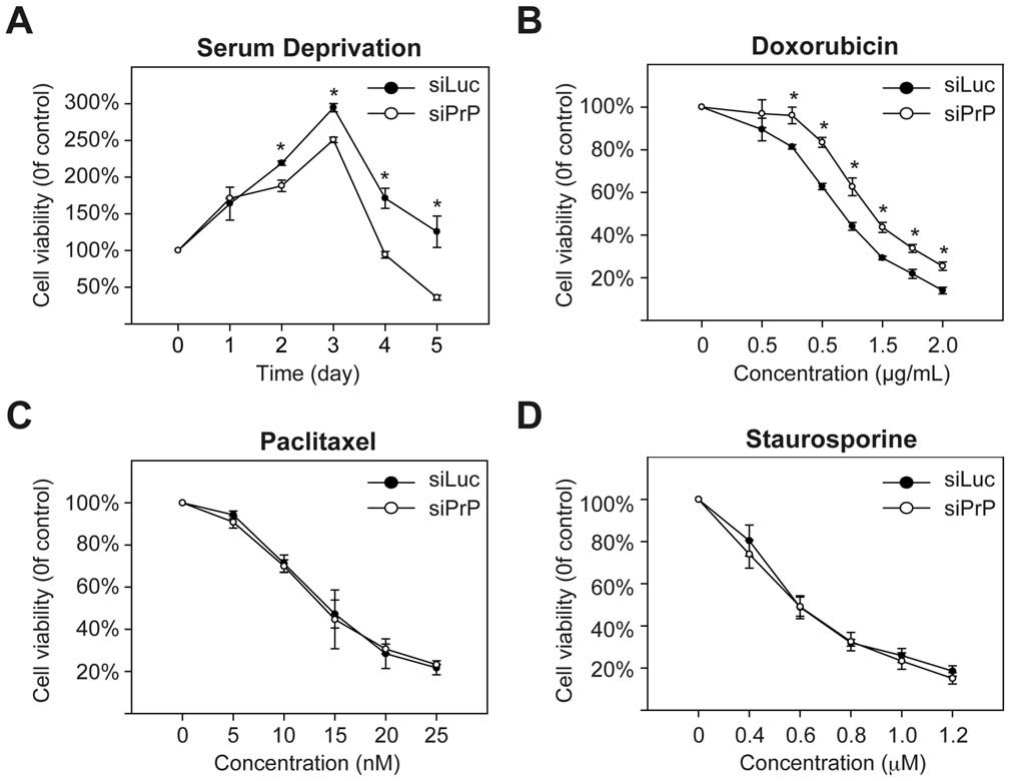
Responses of siPrP and siLuc cells to different cytotoxic stimuli. Cells were subjected to serum deprivation for 5 days (**A**), or incubated with doxorubicin (**B**), paclitaxel (**C**), or staurosporine (**D**) for 48 hours. Cell viability was measured by the MTT assay and the viability of cells without any treatment was set as 100%. Asterisks represent statistically significant differences, which were determined by student's t test and indicated by * P<0.05.

It is well established that PrP-null neurons are much more susceptible to serum deprivation induced cell death [20], [21]. To determine whether PrP has a similar protective function in breast cancer cells, we performed serum deprivation analysis. Both siPrP and siLuc cells continued to grow for ∼3 days in the absence of serum and a significant loss of cell viability was observed on day 4 and 5 of serum deprivation (Fig. 2A). Importantly, the PrP knockdown siPrP cells exhibited a significantly lower viability starting from day 2 of serum deprivation (comparing day 2, 3, 4, and 5 on Fig. 2A). Thus, we concluded that, similar to its role in neurons [20], [21], PrP protects MDA-MB-435 cells against serum deprivation induced cytotoxicity.

To determine whether PrP has a similar protective effect against drug induced cell death, we treated siPrP and siLuc cells with three different drugs: the microtubule-stabilizing chemotherapy drug paclitaxel, the general protein kinase inhibitor staurosporine, and the DNA-interacting chemotherapy drug doxorubicin. Our results revealed that PrP knockdown in MDA-MB-435 cells did not affect paclitaxel- or staurosporine-induced cell death (Fig. 2C and D). Surprisingly, we found that PrP knockdown rendered MDA-MB-435 cells more resistant to doxorubicin (Fig. 2B), indicating that PrP expression in MDA-MB-435 cells exacerbates doxorubicin-induced cytotoxicity.

**Figure 3:**
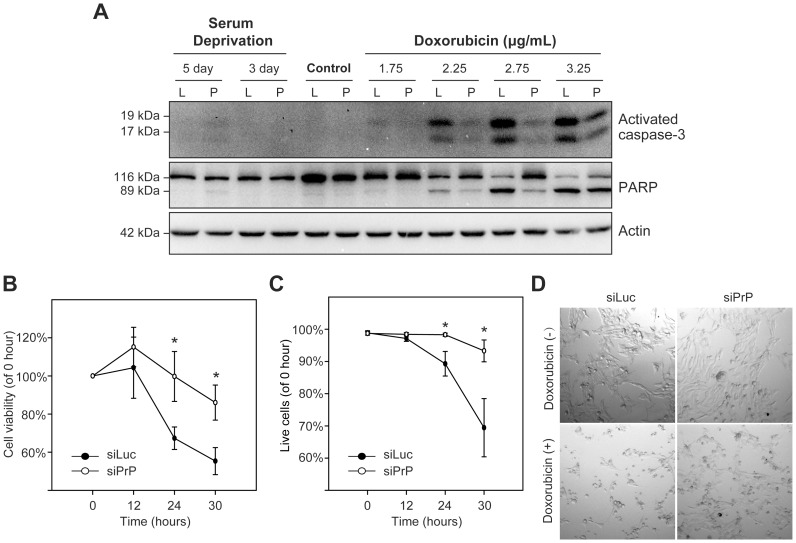
The influence of PrP knockdown on serum deprivation and doxorubicin-induced cytotoxicity. (**A**) Immunoblot analysis of activated caspase-3 and PARP cleavage in siPrP (P) or siLuc (L) cells subjected to serum deprivation for indicated time or doxorubicin treatment for 24 hours at indicated concentrations. Equal loading was verified by immunoblot analysis with an anti-actin antibody. Control, no treatment; L, siLuc cell line; P, siPrP cell line. (**B**) Results of MTT assay showing cell viability after incubation with 1.25 µg/mL doxorubicin for indicated time. (**C**) Live/dead cell number ratio determined by trypan blue exclusion assay after incubation with 1.25 µg/mL doxorubicin for indicated time. Asterisks represent statistically significant differences, which were determined by student's t test and indicated by * P<0.05. (**D**) Representative images of siLuc or siPrP cells incubated without or with 1.25 µg/mL doxorubicin for 30 hours.

The opposite effects of PrP knockdown on serum deprivation- and doxorubicin-induced toxicity were unexpected, particularly the enhanced resistance to chemotherapy drug doxorubicin. To confirm this observation, we analyzed classic markers of cell death, caspase-3 activation and poly ADP ribose polymerase (PARP) cleavage. Faint activated caspase-3 bands (17 and 19 kDa) and a faint cleaved PARP band (89 kDa) were detected in 5-day serum-deprived siPrP cells, but not in siLuc cells (Fig. 3A). In contrast, in cells treated with different concentrations of doxorubicin, the dosage-dependent caspase-3 activation and PARP cleavage were much more reduced in PrP knockdown siPrP cells (Fig. 3A). These observations are consistent with our cytotoxicity results, showing that PrP knockdown in MDA-MB-435 cells has opposite effects on serum deprivation and doxorubicin induced cell death.

The unexpected effect of PrP knockdown on doxorubicin-induced cytotoxicity was further confirmed by a time course study. Both MTT cell viability assay and cell death specific trypan blue exclusion assay confirmed that PrP knockdown attenuated the time-dependent doxorubicin-induced cytotoxicity in MDA-MB-435 cells (Fig. 3B and C). Moreover, the effect of PrP knockdown can also be directly observed by comparing cell morphologies under the microscope (Fig. 3D), which clearly showed a better survival of siPrP cells than the control siLuc cells. Collectively, our results support that stably knocking down PrP in MDA-MB-435 cells increases the resistance to doxorubicin-induced cytotoxicity.

**Figure 4:**
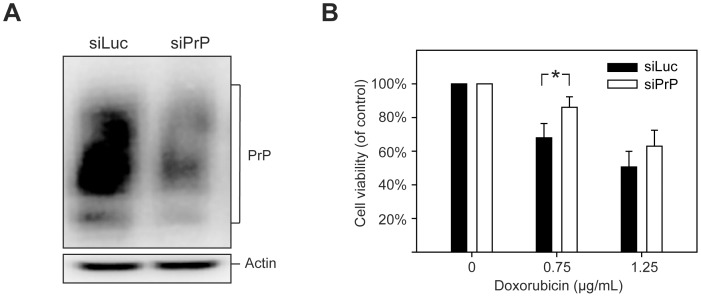
The influence of transient PrP knockdown on doxorubicin induced cytotoxicity in MDA-MB-435 cells. (**A**) Immunoblot analysis of PrP in MDA-MB-435 cells 24 hours after transient transfection with pSIREN-RetroQ-siLuc (siLuc) or pSIREN-RetroQ-siPrP (siPrP) plasmid. (**B**) Twenty-four hours after transient transfection with pSIREN-RetroQ-siLuc (siLuc) or pSIREN-RetroQ-siPrP (siPrP) plasmid, cells were incubated with doxorubicin for 48 hours at indicated concentrations. Cell viability was measured by the MTT assay. Asterisks represent statistically significant differences, which were determined by student's t test and indicated by * P<0.05.

Establishing a stable cell line is always accompanied with the risk of selecting unwanted genetic variations, which is the reason why we used bulk- instead of single colony-selection. To completely rule out this potential caveat, we transiently transfected MDA-MB-435 cells with pSIREN-RetroQ-siPrP or pSIREN-RetroQ-siLuc plasmid. The effect of PrP knockdown was confirmed by immunoblot analysis (Fig. 4A), revealing that PrP protein level was reduced, but the knockdown was not as efficient as that in the selected stable line (Fig. 1A). Nonetheless, after doxorubicin treatment, the viability of PrP knockdown siPrP cells was higher than that of control siLuc cells and the difference at 0.75 µg/mL doxorubicin treatment is statistically significant (Fig. 4B). Together, these results established that PrP knockdown in MDA-MB-435 breast cancer cells increase the resistance to doxorubicin-induced cytotoxicity.

### Changes in Bcl-2 family proteins generally correlate with cytotoxicity

**Figure 5:**
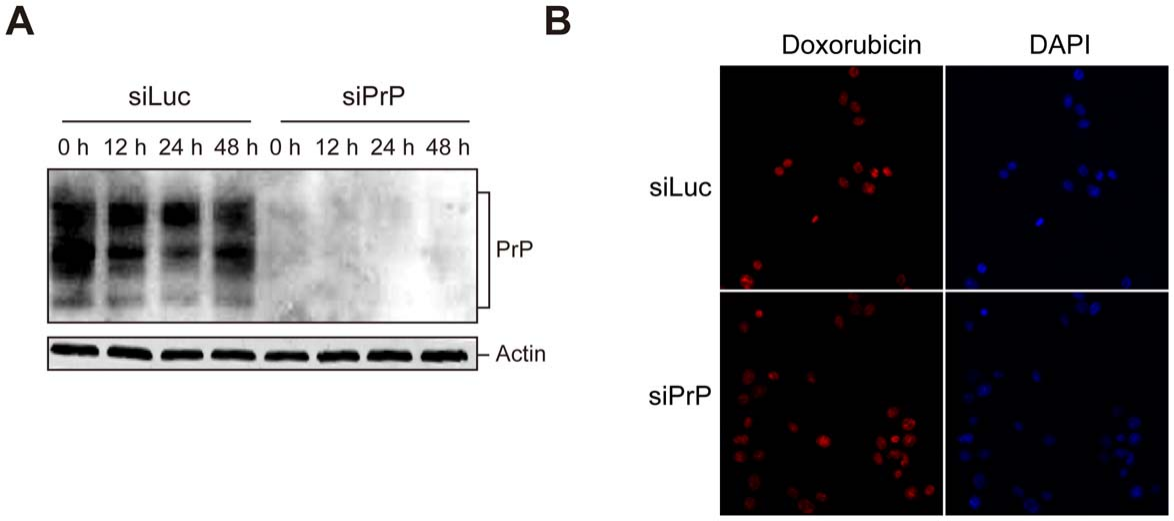
PrP expression level and endogenous fluorescence of doxorubicin during and after doxorubicin treatment. (**A**) Immunoblot analysis of the PrP expression level in siLuc and siPrP cells after incubated with 1.25 µg/mL doxorubicin for indicated time. (**B**) The endogenous fluorescence of doxorubicin after incubating with 1.25 µg/mL doxorubicin 30 hours. Nuclei were stained with DAPI as indicated.

Several analyses were performed to understand the cellular mechanism of PrP-mediated alteration in doxorubicin-induced cytotoxicity. Since PrP promoter contains a heat-shock element (HSE) and PrP is up-regulated under certain stress conditions [22], [23], [24], it is possible that doxorubicin treatment induces PrP expression that somehow overcomes shRNA suppression. We ruled out this possibility by determining PrP protein levels in doxorubicin-treated siPrP cells, which did not show any up-regulation (Fig. 5A). Second, PrP is known to interact with P-gp in breast cancer cells [15] and PrP knockdown may lead to a more efficient removal of doxorubicin by P-gp. This possibility was tested by monitoring endogenous fluorescence of doxorubicin under the microscope, which revealed that doxorubicin was located in almost every cell and there was no difference between the PrP knockdown siPrP and the control siLuc cells (Fig. 5B).

**Figure 6:**
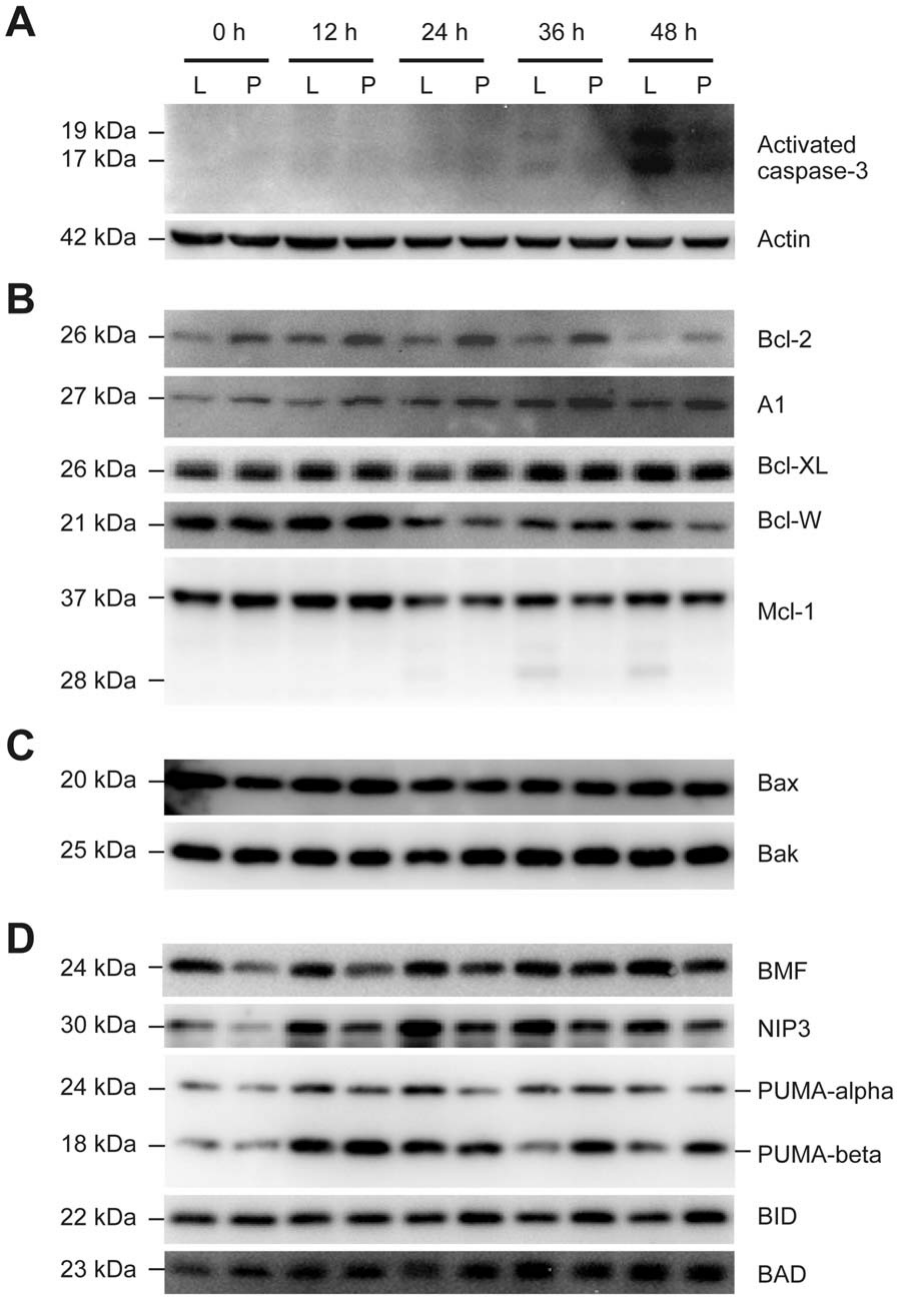
Changes of Bcl-2 family proteins in siPrP and siLuc cells after doxorubicin treatment. Immunoblot analysis of activated caspase-3 (**A**) and Bcl-2 family proteins (**B, C, D**) after incubation with 1.25 µg/mL doxorubicin for indicated time. Equal loading was verified by immunoblot analysis with an anti-actin antibody. L, siLuc cells, P, siPrP cells.

Next, we tested the possibility that PrP knockdown may affect Bcl-2 family protein expression based on previous reports of the PrP-Bcl-2 family connection [6], [14]. Activated caspase-3 was used as an indication of cell death, which was activated at later time points of doxorubicin treatment and the level of caspase-3 activation was reduced in siPrP cells (Fig. 6A).

Bcl-2 family is divided into 3 groups: the anti-apoptotic members (Fig. 6B), the pro-apoptotic members (Fig. 6C), and the pro-apoptotic BH3-domain-only members (Fig. 6D). Our results showed that PrP knockdown led to a slight increase in anti-apoptotic Bcl-2 and A1 expression (Fig. 6B, 0 hour) and a slight decrease in pro-apoptotic member BMF and NIP3 expression (Fig. 6D, 0 hour).

Doxorubicin treatment caused a variety of changes (Fig. 6 B–D, 12–48 hour). For anti-apoptotic members (Fig. 6B), Bcl-2 was first induced and then decreased, A1 was increased, Bcl-XL did not show obvious change, while Mcl-1 and Bcl-W were decreased gradually. The smaller 28 kDa Mcl-1 fragment observed in siLuc cells at later time points was a known cleavage product that has pro-apoptotic function [25]. In general, the doxorubicin-induced changes in anti-apoptotic bcl-2 members were consistent with cytotoxicity results. Except for the slight increase of A1, three members of this group, Bcl-2, Bcl-XL, and Mcl-1, were all reduced at later time points.

For the pro-apoptotic members (Fig. 6C) and pro-apoptotic BH3-domain-only members (Fig. 6D), Bax, Bak, and BID expression did not change significantly, NIP3, PUMA-alpha and PUMA-beta expression were first increased and then decreased gradually after doxorubicin treatment, and the expression of BMF or BAD was increased. Thus, doxorubicin-induced changes in pro-apoptotic Bcl-2 proteins were also generally consistent with cytotoxicity results. Except for three unchanged proteins, the majority of pro-apoptotic Bcl-2 proteins tested here were increased at some time points during the course of doxorubicin treatment.

The change of Bcl-2 family proteins in PrP knockdown siPrP cells was also consistent with its increased survival. The increased Bcl-2 and A1 expression and the decreased pro-apoptotic BMF and NIP3 expression were maintained even at later time points of doxorubicin treatment (Fig. 6B and D). The detection of the pro-apoptotic 28 kDa Mcl-1 fragment only in the control siLuc cells correlates with its higher toxicity. The only protein seemed to be different from cytotoxicity results was PUMA-beta, which increased in siPrP cells at 36- and 48-hour time points (Fig. 6D, PUMA-beta). Since multiple studies showed that PUMA-alpha, but not PUM-beta, is involved in chemically induced apoptosis and PUMA-beta expression does not correlate with cell death in some cases [26], [27], this result is still consistent with cytotoxicity results. Together, these changes in Bcl-2 family proteins may explain the increased resistance to doxorubicin in siPrP cells. However, alterations in multiple Bcl-2 family proteins suggest that these changes cannot be the direct link between PrP and doxorubicin-induced cytotoxicity. Changes in other signaling molecules upstream of Bcl-2 likely play a more direct role.

### The effect of PrP on doxorubicin-induced cytotoxicity is independent of p53

Doxorubicin is a DNA damaging compound activating p53 pathway. It has been shown that doxorubicin-induced toxicity in cardiomyocytes involves ERK/p53 signaling pathways [28]. Moreover, PrP has been shown to enhance neuronal cells' sensitivity to staurosporine-induced cell death in a p53-dependent manner [29], [30]. Thus, we investigated the possible influence of p53 on PrP-mediated enhancement of doxorubicin-induced cytotoxicity in breast cancer cells.

**Figure 7:**
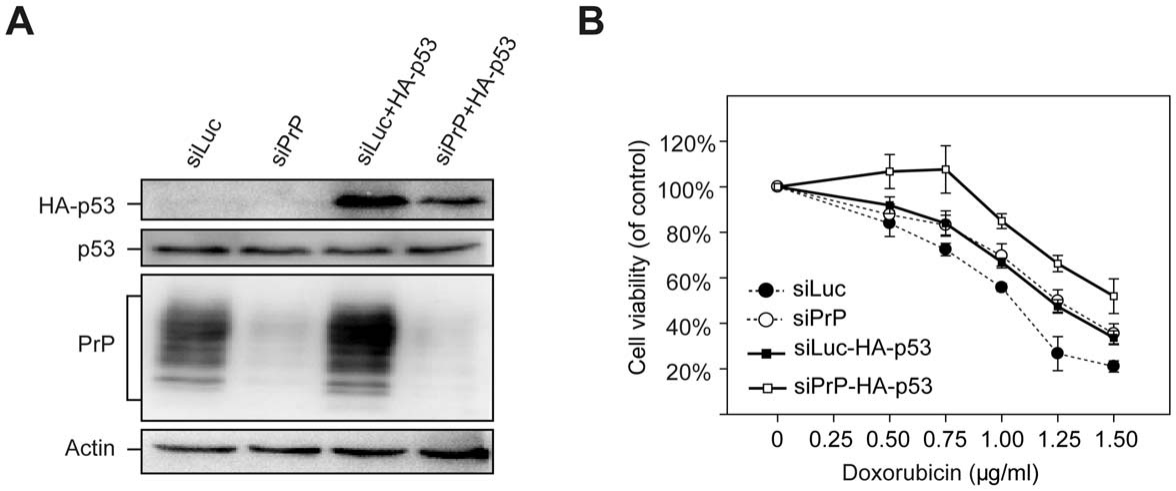
Effects of p53 on doxorubicin-induced cytotoxicity in siLuc and siPrP cells. (**A**) Immunoblot analyses of transfected wild-type HA-p53 expression (anti-HA antibody), total p53 expression (anti-p53 antibody), and PrP expression (anti-PrP antibody). Immunoblot analysis of actin was performed to verify the total input of cell lysates. (**B**) After incubation with doxorubicin for 48 hours at indicated concentrations, cell viability was measured by the MTT assay. Differences between siLuc with siPrP were statistically significant (p<0.5, student's t test) at doxorubicin concentrations of 0.75, 1.00, 1.25 and 1.50 µg/mL. Differences between siLuc-HA-p53 with siPrP-HA-p53 were statistically significant (p<0.5, student's t test) at all doxorubicin concentrations.

Using a luciferase reporter assay, we first confirmed that the loss-of-function p53 mutation in MDA-MB-435 cells [31] was maintained in our stable lines (data not shown), indicating that the observed difference in doxorubicin-induced cytotoxicity is independent of p53 transcriptional function. Next, we stably transfected siPrP and siLuc cells with a functional HA-tagged p53 (HA-p53) and named the cell lines as siPrP-HA-p53 and siLuc-HA-p53, respectively. Characterization of these cells revealed that HA-p53 was expressed (Fig. 7A) and functional as a transcription factor (using the luciferase reporter assay, data not shown), and a functional HA-p53 did not alter PrP expression in these cells (Fig. 7A). When these cells were treated with doxorubicin, cells stably expressing HA-p53 were more resistant to doxorubicin-induced cytotoxicity, supporting that HA-p53 is functional in these cells (Fig. 7B). However, the difference in cytotoxicity caused by PrP knockdown remained (Fig. 7B, comparing siLuc with siPrP, and siLuc-HA-p53 with siPrP-HA-p53). Together, these results suggest that the PrP knockdown induced doxorubicin resistance is independent of p53.

### The effect of PrP on doxorubicin-induced cytotoxicity involves ERK pathway

**Figure 8:**
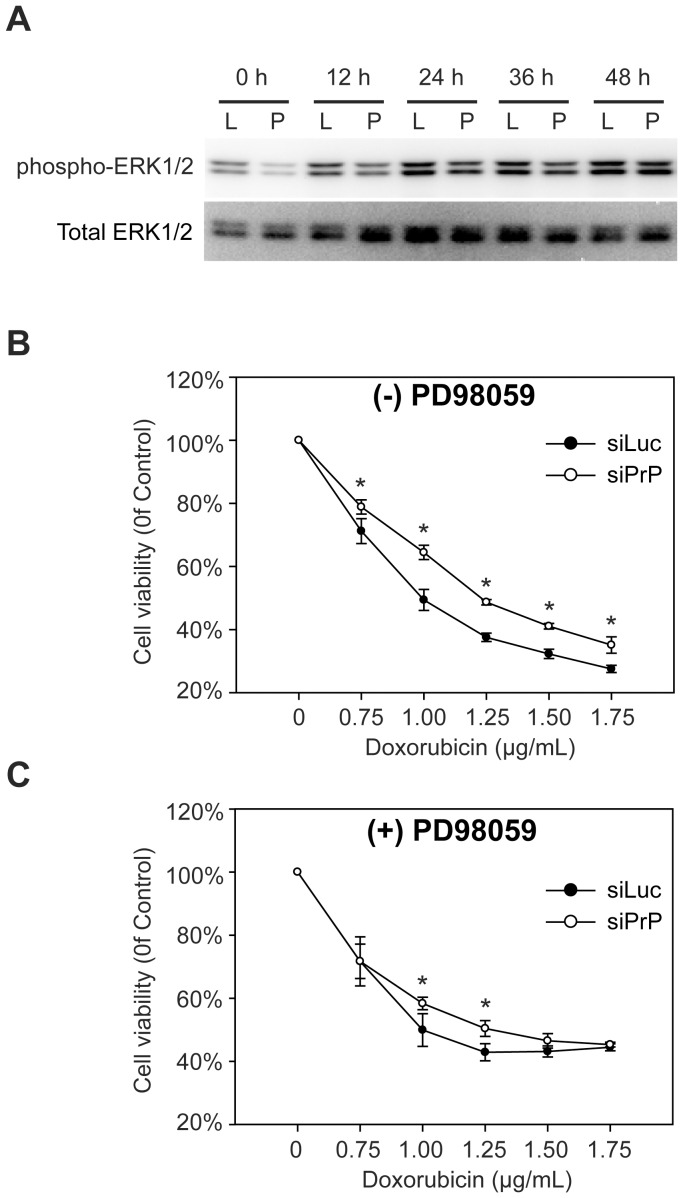
ERK signaling pathway is involved in PrP knockdown induced resistance to doxorubicin. (**A**) Immunoblot analysis of the phosphorylated ERK1/2 (phospho-ERK1/2) and total ERK1/2 after incubation with 1.25 µg/mL doxorubicin for indicated time. L, siLuc cells; P, siPrP cells. Cells without (**B**) or with (**C**) pre-incubation with PD98059 were incubated with doxorubicin for 48 hours at indicated concentrations and cell viability was measured by the MTT assay. Asterisks represent statistically significant differences, which were determined by student's t test and indicated by * P<0.05.

The ERK signaling pathway was first investigated by immunoblot analysis. Figure 8A showed that phosphorylated ERK1/2 was slightly reduced in PrP knockdown siPrP cells (Fig. 8A, 0 hour). Doxorubicin treatment led to an increase of phosphorylated ERK1/2, indicating the activation of ERK signaling pathway. More importantly, the level of phosphorylated ERK1/2 in siPrP cells remained lower at almost all the time points (Fig. 8A). Similar results were obtained by treating these cells with various dosages of doxorubicin (data not shown).

To determine whether the difference in ERK activation contributes to the effect associated with PrP knockdown, we used MEK inhibitor PD98059 to block ERK1/2 phosphorylation. Cells pre-incubated with or without PD98059 were treated with various concentrations of doxorubicin and cell viability was measured. The PrP knockdown induced doxorubicin resistance was clearly detected in cells without PD98059 pre-incubation (Fig. 8B). However, in cells pre-incubated with PD98059, the difference in cytotoxicity was greatly reduced and no statistically significant difference was detected in cells treated with 3 out of 5 doxorubicin concentrations (Fig. 8C). These results suggest to us that ERK signaling pathway is at least partially responsible for PrP knockdown induced doxorubicin resistance.

## Discussion

Previous studies of PrP in breast cancer are generally consistent with the notion that PrP expression increases the resistance to cytotoxicity [11], [15], [16], [17], [18], indicating that reducing PrP expression could potentially enhance the efficiency of chemotherapy. Our study intended to test whether PrP knockdown is able to enhance chemotherapeutic drug induced toxicity in breast cancer cells without pre-selection. Surprisingly, we found that PrP knockdown in MDA-MB-435 cells not only fails to enhance the susceptibility, but also increases the resistance to doxorubicin-induced cytotoxicity. This finding, although unexpected, is generally consistent with a previous study by Meslin et al. on PrP knockdown in doxorubicin-resistant MCF7 sublines. They showed that PrP knockdown only enhances the susceptibility to TRAIL-, but not to doxorubicin-induced cytotoxicity [17]. The use of doxorubicin-resistant cell lines may prevent them to detect the increased resistance to doxorubicin. Nevertheless, our findings that PrP knockdown in MDA-MB-435 cells leads to opposite responses to cytotoxic stimuli suggest that the role of PrP in breast cancer biology is complicated, which may vary among different types of cancer cells. Further studies are required to determine whether manipulating PrP expression can be a valid approach to benefit cancer treatment.

Our results indicate that reduced phosphorylated ERK1/2 in PrP knockdown siPrP cells is partially responsible for PrP-mediated susceptibility to doxorubicin. Depending on cell type and cytotoxic stimuli, ERK signaling pathway can increase cell survival or cell death [32], [33]. A sustained activation of ERK (6 to 72 hours) has been observed in most of cell deaths involving ERK pathway, including several doxorubicin-induced cell death [32]. Interestingly, a sustained ERK activation was observed in our study (Fig. 8, ERK activation >48 hours), indicating that ERK activation is involved in the doxorubicin-induced cell death process in our experimental system. The pro-cell death activity of activated ERK would account for the reduced cytotoxicity in siPrP cells.

The opposite roles of PrP in cell death induced by serum deprivation and doxorubicin are intriguing, which might be attributed to the very different cellular mechanisms responsible for these two types of cytotoxicity. For example, the transcription factor hypoxia-inducible factor-1α (HIF-1α) is induced during serum deprivation and plays an important role in cell survival [34]. Yet, doxorubicin is a potent inhibitor of HIF-1-mediated gene transcription [35]. PrP knockdown in MDA-MB-435 cells may alter multiple signaling pathways. Depending on which pathway is involved in the cellular response to a particular cytotoxic stimulus, PrP knockdown may have pro- or anti-cell death effect.

Thus far, the physiological function of PrP remains unclear. Regarding the role of PrP in cell death and survival, most previous studies indicate a pro-survival function through different cellular mechanisms [6], [14]. Notably, a few studies did reveal a pro-cell death function of PrP under certain conditions. Paitel et al. reported that, in TSM1 neuronal cells and primary cultured neurons, PrP enhances staurosporine-induced toxicity by regulating p53 pathway at both transcriptional and post-transcriptional levels [29], [30]. In a more recent study, Anantharam et al. reported that PrP plays a pro-apoptotic role during endoplasmic reticulum (ER) stress in neuronal cells [36]. Interestingly, they also found that PrP protects cells against oxidative-stress induced cell death in the same cell line. Our study showed the first example of opposite effects of PrP on cell survival in a non-neuronal cell line. More importantly, the cytotoxic stimuli used in our study were serum deprivation and doxorubicin, which are very different from ER stress and oxidative stress used in the study by Anantharam et al [36]. In addition, we have shown that the pro-cell death effect of PrP on doxorubicin-induced cell death is clearly independent of p53, which is different from the mechanism suggested by Paitel et al. [29], [30]. These interesting observations suggest to us that the alteration of Bcl-2-like cell death signaling molecule in PrP knockdown cells is most likely an indirect consequence of PrP silencing. Real PrP function likely involves a cellular process that has a global impact on cell physiology and PrP knockdown would result in various changes in multiple signaling pathways. Depending on the specific changes in the pathway associated with a particular cytotoxic stimulus, PrP silencing could result in pro- or anti-cell death effect. This mechanism would also account for the apparent lack of effect on cell death caused by paclitaxel or staurosporine (Fig. 2). The pathways upstream of Bcl-2-like cell death signaling molecule are specified by a particular cytotoxic stimulus, which may or may not affected by PrP knockdown. In paclitaxel or staurosporine induced cell death in MDA-MB-435 cells, the upstream pathways are probably not affected by PrP knockdown, resulting in no change in cell death observed in our study.

Collectively, our study revealed that PrP knockdown in human breast cancer MDA-MB-435 cells results in pleiotropic responses to various cytotoxic stimuli, which is likely due to PrP knockdown induced alterations in multiple cellular signaling pathways. These findings may have implications not only for understanding of the role of PrP in breast cancer biology, but also for elucidating the physiological function of PrP.
